# The nuclease FAN1 is involved in DNA crosslink repair in *Arabidopsis thaliana* independently of the nuclease MUS81

**DOI:** 10.1093/nar/gkv208

**Published:** 2015-03-16

**Authors:** Natalie J. Herrmann, Alexander Knoll, Holger Puchta

**Affiliations:** Botanical Institute II, Karlsruhe Institute of Technology, Hertzstrasse 16, Karlsruhe, 76187, Germany

## Abstract

Fanconi anemia is a severe genetic disorder. Mutations in one of several genes lead to defects in DNA crosslink (CL) repair in human cells. An essential step in CL repair is the activation of the pathway by the monoubiquitination of the heterodimer FANCD2/FANCI, which recruits the nuclease FAN1 to the CL site. Surprisingly, FAN1 function is not conserved between different eukaryotes. No FAN1 homolog is present in *Drosophila* and *Saccharomyces cerevisiae*. The FAN1 homolog in *Schizosaccharomyces pombe* is involved in CL repair; a homolog is present in Xenopus but is not involved in CL repair. Here we show that a FAN1 homolog is present in plants and it is involved in CL repair in *Arabidopsis thaliana*. Both the virus-type replication-repair nuclease and the ubiquitin-binding ubiquitin-binding zinc finger domains are essential for this function. FAN1 likely acts upstream of two sub-pathways of CL repair. These pathways are defined by the Bloom syndrome homolog RECQ4A and the ATPase RAD5A, which is involved in error-free post-replicative repair. Mutations in both FAN1 and the endonuclease MUS81 resulted in greater sensitivity against CLs than in the respective single mutants. These results indicate that the two nucleases define two independent pathways of CL repair in plants.

## INTRODUCTION

Fanconi anemia (FA) is an inherited disease in humans with symptoms such as bone marrow failure, congenital abnormalities and cancer. On a cellular level, all FA patients show the following characteristic feature that can be used for diagnostics: affected cells exhibit an increased sensitivity against interstrand crosslink (CL)-inducing agents such as mitomycin C (MMC). FA was first described in 1927 by the Swiss pediatrician Dr Guido Fanconi (1). The disorder is linked to a mutation in one of 17 currently known *FANC* genes. All *FANC* genes are part of a common pathway, known as the FA pathway, to repair interstrand CLs. After the appearance of an interstrand CL, the FANC protein FANCM and its interacting partners MHF1, MHF2 and FAAP24 recognize and bind to the lesion. This binding leads to the recruitment of the so-called FA core complex. The core complex consists of seven FANC proteins (FANCA, -B, -C, -E, -F, -G and -L). If activated, the complex is able to monoubiquitinate and activate a heterodimer formed by FANCI and FANCD2; this heterodimer is known as the FANCI/FANCD2 (ID) complex (2). Due to this monoubiquitination, the ID complex is recruited to the DNA damage site. There, it facilitates the activation and recruitment of downstream *FANC* genes to repair the lesion. In addition to the *FANC* genes, several other FA-associated proteins have been described that are essential for CL repair (3,4). One of those FA-associated factors is the nuclease FAN1 (Fanconi/FANCD2 associated nuclease I), the recruitment of which is dependent on the monoubiquitination of FANCD2 (5–8). Mutations in *FAN1* in humans are linked to an increased sensitivity against interstrand CL-inducing agents. In genome-wide association studies, mutations in the *FAN1* gene in humans have been associated with chronic kidney failure (9), schizophrenia and autism (10). However, no patient with FA has been diagnosed with causal mutations in *FAN1*, so that FAN1 is discussed as a FA-associated protein. A crystal structure of the FAN1 protein was recently elucidated (11). FAN1 is composed of a ubiquitin-binding zinc finger (UBZ) domain at the N-terminus, a central SAF-A/B, Acinus and PIAS (SAP) domain and a virus-type replication-repair (VRR) nuclease domain at the C-terminus. Through the UBZ domain, FAN1 is able to interact with monoubiquitinated FANCD2 and localize to stalled replication forks (5–8,12). The UBZ domain directly binds to ubiquitinated proteins; this binding is essential for FAN1 function (12). The SAP domain is involved in DNA binding. The VRR nuclease domain mediates 5′-3′-exonuclease and endonuclease activity. It has been shown that human FAN1 cleaves DNA successively at every third nucleotide (13). The current model of interstrand CL repair during S-phase integrates translesion synthesis, nucleotide excision repair and homologous recombination (HR) (14). However, the initial step in this repair is an incision via endonucleases to unhook the CL. Two nuclease complexes, MUS81-EME1 and XPF-ERCC1, have been postulated to catalyze this unhooking step (15–18). It was shown that both complexes cut 3′ flaps *in vitro*; this is the opposite polarity of the FAN1 nuclease. It has been postulated that FAN1 is also involved in the unhooking step; this protein may act on the other side of the CL as one of the two nuclease complexes, MUS81-EME1 or XPF/ERCC1 (19). FAN1 might also be involved in HR during interstrand CL repair by processing 3′ ends at the beginning of HR or functioning at a later step (5,7–8).

In addition to functions during the repair of interstrand CLs, HsFAN1 is also involved in the regulation of mitosis. FAN1 is degraded during the end of mitosis and is likely regulated by the APC/C-complex (anaphase-promoting complex/cyclosome) (20), the main regulator during mitosis. The FA protein FANCD2 is not expressed during mitosis, suggesting that FAN1 might take on functions independently of the FA pathway (20,21).

The FAN1 protein seems to not be conserved in all organisms; no homologs were found in *Drosophila* or *Saccharomyces cerevisiae* (8). In *Schizosaccharomyces pombe*, a homolog of FAN1 was identified; SpFan1 is also involved in the repair of interstrand CLs (22). SpFan1 possesses a VRR nuclease domain and a DNA-binding domain. A homolog of FAN1 was also identified in Xenopus. However, this protein seems to have no direct functions in interstrand CL repair; *FAN1* depletion does not affect the repair of this type of damage (23). Furthermore, an additional mutation in the nuclease *MUS81* did not alter the repair of interstrand CLs. However, Douwel *et al*. did not eliminate the possibility that FAN1 is involved in unhooking interstrand CL; FAN1 may act redundantly to another endonuclease (23).

Recently, the FANC protein FANCM and the FA-associated factor MHF1 were characterized in *Arabidopsis thaliana*. Single mutants were not sensitive against interstrand CL-inducing agents (24,25). However, an analysis of the corresponding double mutants of *fancm* or *mhf1* and the RecQ helicase *recq4A* uncovered hidden functions for AtFANCM, AtMHF1 and AtRECQ4A in interstrand CL repair. Moreover, it could be demonstrated that AtFANCM and AtMHF1 act together in a pathway independent of AtRECQ4A (22).

AtRECQ4A belongs to the family of RecQ helicases, which are involved in different cellular processes to ensure genome stability. Mutations in the RecQ helicases BLM, WRN or RECQ4 in humans are associated with the hereditary diseases Bloom syndrome, Werner syndrome and Rothmund-Thomson syndrome, respectively (26–29). It was previously demonstrated that RECQ4A in *A. thaliana* is the functional homolog of the human BLM and yeast Sgs1 helicases (30). Furthermore, it was shown that AtRECQ4A is involved in the repair of methylated bases, intrastrand CLs and interstand CLs that are induced by the genotoxins MMS, *cis*-Platin and MMC, respectively (24,31–32).

The endonuclease MUS81 and the ATPase RAD5A are also involved the repair of interstrand CLs in Arabidopsis; these proteins act in independent pathways (31).

The endonuclease MUS81 forms a nuclease complex with its interacting partner EME1. This complex is involved in the resolution of different DNA intermediates, such as stalled replication forks, displacement loops and Holliday junctions (33–35). In *A. thaliana*, a homolog of MUS81 is part of an active endonuclease (36) and involved in the repair of different types of DNA damage caused by the genotoxins *cis*-Platin, MMC and MMS (37). It was also reported that a double mutant of At*recq4A* and At*mus81* shows synthetic lethality, leading to the hypothesis that RECQ4A and MUS81 act in two independent pathways to resolve stalled replication forks (31).

The ATPase Rad5 is a member of the SNF2 family of proteins and is involved in the post-replicative repair (PRR) pathway in yeast. Furthermore, it was shown that Rad5 in yeast promotes the regression of stalled replication forks (38). In *A. thaliana*, the homolog RAD5A is involved in the repair of DNA damage (39). A mutation in At*RAD5A* resulted in an increased sensitivity against intra- and interstrand CLs induced by *cis*-Platin and MMC and against replication-associated damage caused by MMS (39). A double mutant of At*mus81* and At*rad5A* revealed that both proteins act in two parallel interstrand CL repair pathways (31).

Here, we identify a FAN1 homolog in *A. thaliana* and define its role in DNA repair. We unambiguously demonstrate that FAN1 functions in the repair of interstrand CLs in Arabidopsis. Moreover, complementation approaches revealed that both the VRR nuclease domain and the UBZ domain are essential for this repair function. Using double mutants, we were able to obtain detailed insights into the specific function of FAN1 in the complex network of CL repair pathways in plants.

## MATERIALS AND METHODS

### Plant materials and growth conditions

For the characterization of *FAN1* in *A. thaliana*, the mutant line *fan1-1* (GABI_815C08) from the GABI-Kat collection was used (40). For the generation of double mutants, the mutant lines *mus81-1* (GABI_113F11), *rad5A-2* (SALK_047150), *recq4A-4* (GABI_203C07) and *mhf1-1* (SALK_119435) from the SALK and GABI-Kat collections were used; these lines have been previously described (24,32,37,39,41). To generate double mutants and for genotyping, the plants were grown as previously described (42). Homozygous double mutants were identified in the F2 generation through polymerase chain reaction (PCR)-based genotyping. For this purpose and for the cytological methods, the plants were grown in the greenhouse in soil (1:1 mixture of Floraton 3 and Vermiculite, Deutsche Vermiculite-Dämmstoff GmbH, http://www.vermiculite.de) at 22°C under a 16 h light and 8 h dark cycle. For sensitivity and HR assays, an axenic plant culture was needed. Seeds were surface sterilized with 70% ethanol and 4% sodium hypochlorite and incubated overnight at 4°C for stratification. The seeds were sown on agar plates containing germination medium (GM; 4.9 g/l Murashige & Skoog, including vitamins and MES [2-(N-morpholino) ethanesulfonic acid], 10 g/l sucrose and 7.6 g/l agar (adjusted to pH 5.7 with KOH)). Plates were incubated in a CU-36L4 plant culture chamber (Percival Scientific, Inc., Perry, IA, USA) under stable conditions with 16 h light at 22°C and 8 h dark at 20°C.

### Sensitivity assays

Sensitivity assays were performed as previously described (30). Mutant and wild-type (WT) seeds were sterilized and sown on GM as described above. After one week under standard growth conditions in a CU-36L4 plant culture chamber, plantlets were transferred to six-well plates containing 5 ml of liquid GM for the untreated control and 4 ml of liquid medium for the genotoxin treated samples. The following day, different concentrations of genotoxins were added in 1 ml aliquots. After another 13 days of incubation, the fresh weights of the plantlets were measured. For analysis, the fresh weight was calculated as the fresh weight of the treated plants relative to the fresh weight of the untreated controls from the same line. The genotoxins tested were bleomycin (Selleckchem, http://www.selleckchem.com), *cis*-Platin (Sigma-Aldrich Chemie, http://www.sigmaaldrich.com), hydroxyurea (Sigma-Aldrich Chemie), MMC (Duchefa Biochemie, http://www.duchefa-biochemie.com) and MMS (Sigma-Aldrich Chemie).

### HR assays

The HR assays were performed as recently described (30). For the analysis of the HR frequency, mutant and WT seeds in an IC9 reporter background (43) were sterilized and sown on GM as described above. After one week under standard growth conditions, 50 plantlets from each line were transferred to halved Petri dishes containing 10 ml of liquid GM. The plantlets were grown on liquid medium for an additional 7 days. This was followed by GUS staining for 2 days at 37°C. Plant pigments were then extracted overnight at 60°C. To quantify the blue sectors on each plant, a binocular microscope (Stemi DV4, Carl Zeiss, http://www.zeiss.de) was used.

### Propidium iodide assay

To analyze cell death in the root meristem in *fan1-1* and WT plants, the seeds were surface sterilized and sown on GM as described above. After 4 days, plantlets were transferred to six-well plates containing 5 ml of liquid GM medium for the analysis of spontaneous replication damage; plantlets were transferred to six-well plates containing 5 ml liquid GM media containing 2.5 μg/ml MMC to analyze MMC-induced replication damage. Plates were incubated for 18 h at room temperature. The next day, plantlets were washed in liquid GM and transferred to microscope slides. One hundred microliter of 5 μg/ml propidium iodide (PI) solution was added, and the preparation was covered with a cover slip. Analysis of the roots was performed using a confocal laser scanning microscope (LSM700, Carl Zeiss).

### Quantitative real-time expression analysis

To analyze gene expression in the *fan1-1* mutant, total RNA was extracted from two-week-old seedlings using the RNeasy Plant Mini Kit (Qiagen GmbH, http://www.qiagen.com/). As a control, RNA from WT plants was also extracted. cDNA was produced by reverse transcription using an oligo-(dT)_18_ primer and the RevertAid First-Strand cDNA Synthesis Kit (Thermo Fisher Scientific Biosciences GmbH, http://www.thermofisher.com). To analyze gene expression, quantitative real-time PCR was performed (50 cycles of amplification were carried out using the following protocol: 95°C for 10 s, 55°C for 20 s, 72°C for 40 s, detection occurred at the amplification step) with SYBR Green I Master Mix (Roche Diagnostics GmbH, http://www.roche.de). For normalization, the constitutively expressed housekeeping gene *ACTIN2* (AT3G18780) was amplified with the primer pair 5′-CAGATGCCCAGAAGTCTTG-3′ and 5′-GTGCTGTGATTTCTTTGCTC-3′. The primer pair used to analyze the *FAN1* fragment located 5′ from the insertion were as follows: FAN1_RT1_fw 5′-GGATTCTGCTCACGCTGC-3′ and FAN1_RT0_rev 5′-CAATAGTCCCTGCTCTGC-3′. The primers for the analysis across the T-DNA insertion were as follows: FAN1_RT_fw 5′- CAGTGGAGAAGGAGGAGG-3′ and FAN1_RT_rev 5′- CTCCCAAGCCACTCCTCT-3′. To amplify the downstream junction, the following primers were used: FAN1_RT2_fw 5′-GAGGTATGTGTATAGCATCG-3′ and FAN1_RT3_rev 5′- CATTAGAAGTAGAAGCCAAG-3′.

The primer pair FAN1_RT_fw and FAN1_RT_rev that is located 5′ and 3′ of the T-DNA insertion site was also used in qPCR reactions to assess expression of all FAN1 complementation constructs. Here, ACT2 expression was also used for normalization.

### Primers used for PCR-based genotyping of T-DNA insertion lines

To identify plants homozygous for the T-DNA insertion, two primer pairs for each mutant line were used. To detect WT loci, one primer located upstream and one located downstream of the T-DNA insertion were used. To identify T-DNA insertions, one gene-specific primer and one primer located on the T-DNA were used. For *fan1-1*, WT PCR was performed using the following primers: FAN1-2 5′-GCAAAGGCGGATTCTTCG-3′ and FAN1-R2 5′-GAAGCAGGTCTTACTTTGC-3′. For the T-DNA analysis, FAN1-2 and the T-DNA-specific primer LB1 5′-GACCATCATACTCATTGC-3′ were used. Genotyping of the mutant lines *mus81–1, rad5A-2, recq4A-4* and *mhf1-1* was performed as previously described (24,32,37,39).

## RESULTS

### Identification of a single *FAN1* gene in *A. thaliana*

**Figure 1.**
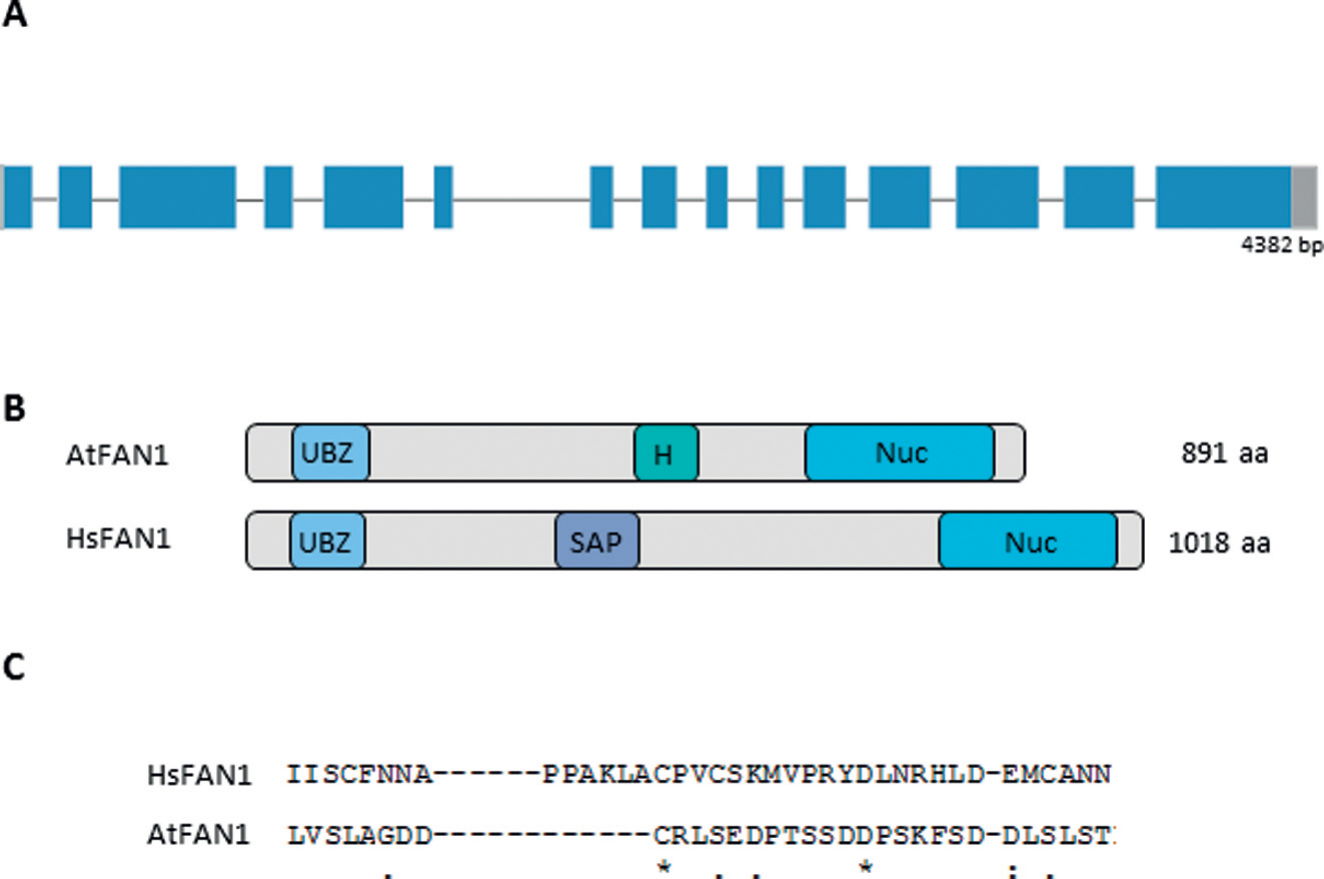
The genomic structure and the protein domains of AtFAN1. (**A**) AtFAN1 is composed of 15 exons and 14 introns with a total length of 4382 bp from the start to stop codons. Exons are shown as blue boxes, and introns are shown as gray lines. UTR regions are shown as gray boxes. (**B**) Domains present in the FAN1 proteins of humans and Arabidopsis. Both contain a VRR nuclease domain (Nuc), a potential UBZ domain and a DNA-binding domain in between [a HIRAN (H) domain in Arabidopsis and a SAP domain in humans]. (**C**) Alignment of HsFAN1 and AtFAN1 protein sequences reveals several conserved amino acids in the UBZ domain region. Identical amino acids are marked by (‘*’), highly similar aa are marked by (‘:’) and aa that are only weakly similar are marked by (‘.’).

**Figure 2.**
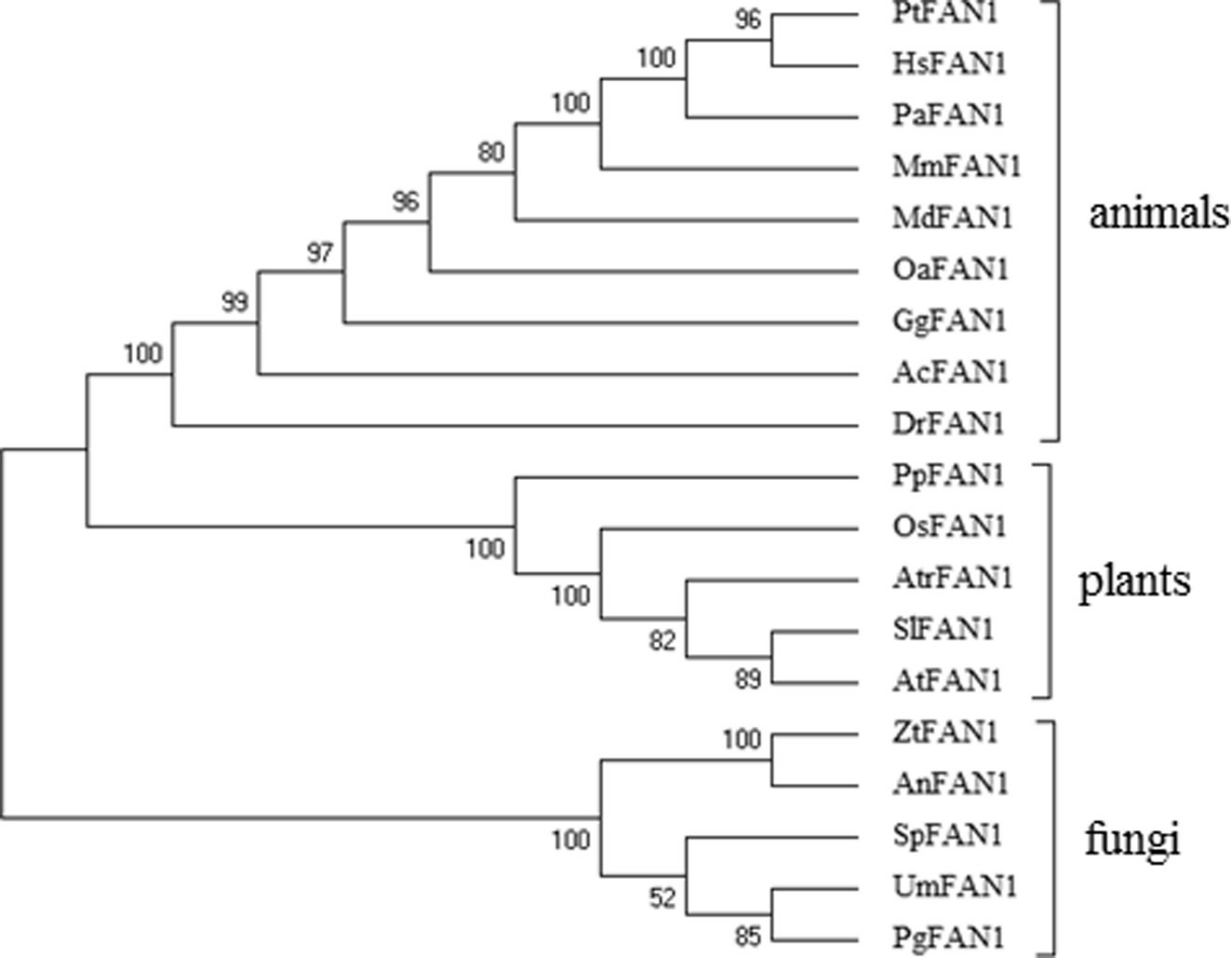
Phylogenetic relationship of FAN1 homologs. Following the alignment of protein sequences by ClustalOmega, a tree was calculated using the maximum likelihood method. The values near the branches represent the percentage of replicate trees in which the associated taxa clustered together in the Bootstrap test (1000 replicates). Pt (*Pan troglodytes*), Hs (*Homo sapiens*), Pa (*Pongo abelii*), Mm (*Mus musculus*), Md (*Monodelphis domestica*), Oa (*Ornithorhynchus anatinus*), Gg (*Gallus gallus*), Ac (*Anolis carolinensis*), Dr (*Danio rerio*), Pp (*Physcomitrella patens*), Os (*Oryza sativa*), Atr (*Amborella trichopoda*), Sl (*Solanum lycopersicum*), At (*Arabidopsis thaliana*), Zt (*Zymoseptoria tritici*), An (*Aspergillus nidulans*), Sp (*Schizosaccharomyces pombe*), Um (*Ustilago maydis*), Pg (*Puccinia graminis*).

A search for homologs of the human *FAN1* gene in *A. thaliana* using BLAST analyses revealed a single hit at the locus AT1G48360. At*FAN1* has a length of 4382 bp between the start and stop codon; the gene is composed of 15 exons and 14 introns (Figure 1A). The predicted protein (Q5XVJ4) has a length of 891 aa and contains a HIRAN domain near its C-terminus (PFAM08797) and a VRR nuclease domain at its N-terminus (PFAM08774) (Figure 1B). The human homolog contains a SAP domain in a similar position as the HIRAN domain. Both of these domains are postulated to be involved in DNA binding. Using domain search algorithms, a UBZ domain could not be detected in AtFAN1; however, protein sequence alignments containing HsFAN1 and AtFAN1 revealed several conserved and similar amino acids in the region containing the human UBZ domain. Therefore, we assume that AtFAN1 might contain a functional UBZ domain (Figure 1C). To determine the evolutionary relationship between different FAN1 homologs, a phylogenetic tree was derived from a ClustalOmega (http://www.ebi.ac.uk/Tools/msa/clustalo/) multiple sequence alignment containing sequences of different FAN1 proteins from animals, plants and fungi. This phylogenetic tree was calculated using the maximum likelihood method in MEGA 6.0 (44) (Figure 2). In general, FAN1 can be found in all plant species tested. Therefore, this protein is likely of some biological importance for plants in general. Within the plant clade, the FAN1 homologs follow the evolutionary relationship of the species analyzed. For example, FAN1 from *A. thaliana* and tomato (*Solanum lycopersicum*) are closely related. These two sequences form a clade with the basal angiosperm *Amborella trichpoda*. FAN1 homologs in rice (*Oryza sativa*) and the moss *Physcomitrella patens* are more distantly related to Arabidopsis FAN1. The phylogenetic relationship is also conserved within animals and fungi. Surprisingly, the relationship between animal and plant FAN1 homologs seems to be closer than the relationship between the animal and fungal FAN1 homologs.

To analyze the function of *FAN1* in *A. thaliana*, we used the following T-DNA insertion line from the GABI-KAT collection: GABI_815C08, here named *fan1-1* (40). Based on the sequence of the insertion site, we determined that the T-DNA is inserted in intron 14 (Supplementary Figure S1A). The insertion site was verified by sequencing, and the 5′ end of the insertion could be characterized in detail (Supplementary Figure S1B). Because the T-DNA insertion site is located within an intron, we tested the expression of *FAN1* in the *fan1-1* mutant line in the regions 5′ and 3′ from and across the T-DNA insertion by quantitative real-time PCR. An expression level similar to WT plants was observed in the region 5′ of the insertion site. Little or no *fan1-1* expression was detected in the regions across and 3′ from the T-DNA insertion site (Supplementary Figure S2).

### AtFAN1 is involved in interstrand CL repair

**Figure 3.**
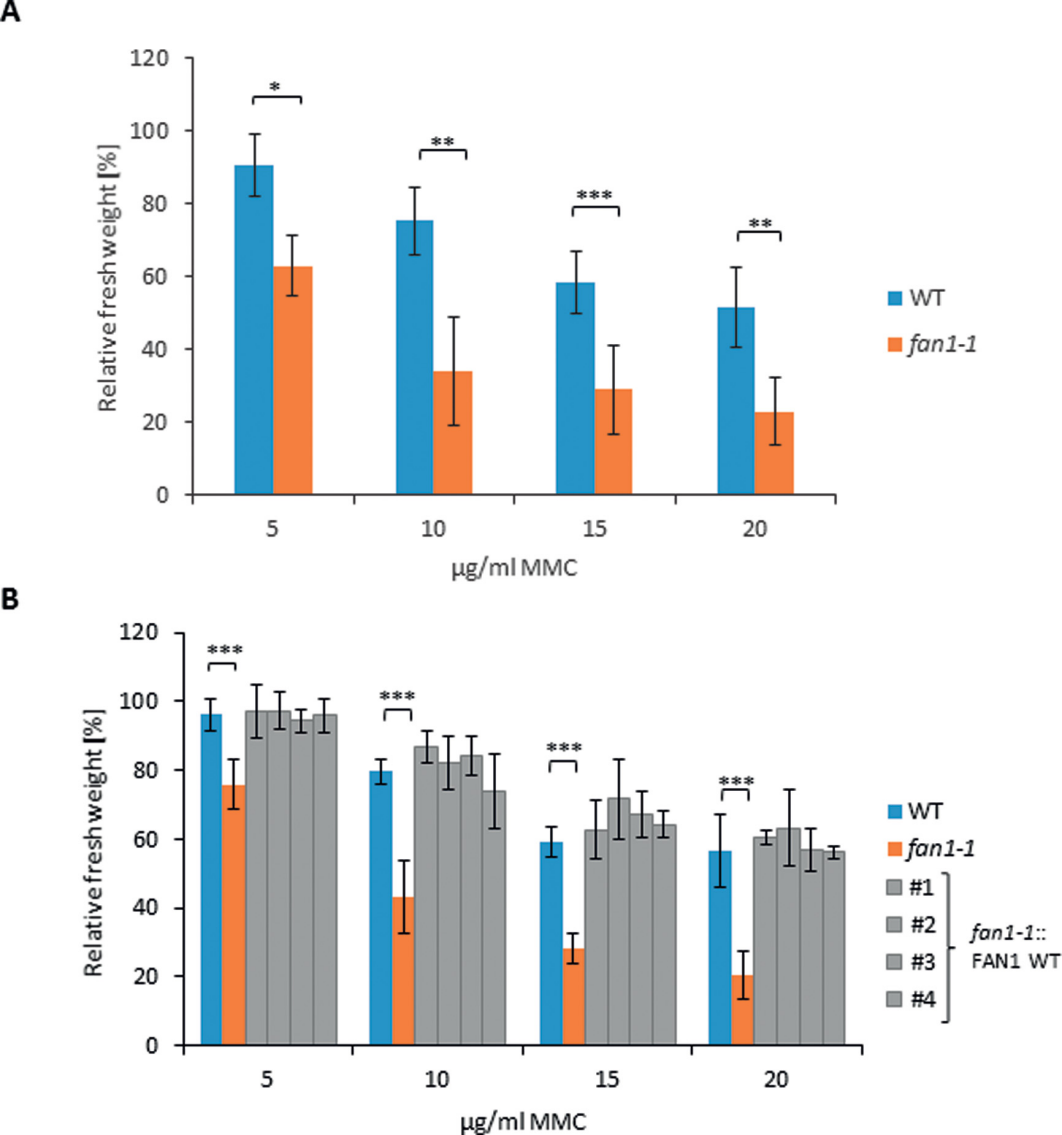
Sensitivity of At*fan1–1* and the *fan1-1*::FAN1 WT complementation lines after MMC treatment. To calculate relative fresh weights of the tested lines, the absolute fresh weights of MMC-treated plants were normalized with fresh weights of untreated control plants from identical lines. Each assay was performed at least three times to calculate mean values and standard deviations (error bars). (**A**) Compared to WT plants, the At*fan1-1* mutant showed a reduced relative fresh weight after MMC treatment. (**B**) The *fan1-1*::FAN1 WT complementation lines #1, #2, #3 and #4 were able to complement the increased sensitivity of *fan1-1* after MMC treatment. *P*-value ≤ 0.05 (*); *P*-value < 0.01 (**); *P*-value < 0.001 (***).

To analyze if FAN1 plays a role in the repair of interstrand CLs in Arabidopsis, we tested the *fan1-1* mutant line for hypersensitivity against the interstrand CL-inducing agent MMC (45). AtFAN1 seems to be involved in the repair of interstrand CLs, as the single mutant showed an increased sensitivity against MMC (Figure 3A). To test whether FAN1 has a more general role in DNA repair in Arabidopsis, we tested the sensitivity of the *fan1-1* mutant against bleomycin, *cis*-Platin, hydroxyurea, methyl methanesulfonate and camptothecin. However, none of these treatments revealed any hypersensitivity (Supplementary Figure S3).

To demonstrate that the observed phenotype of *fan1-1* after MMC treatment is indeed due to a mutation in the *FAN1* gene, we cloned a FAN1 WT construct. This construct included the full-length *FAN1* gene under the control of its natural promoter and terminator. This construct was then transformed into the *fan1-1* mutant; and four genetically independent complementation lines (*fan1-1*::FAN1 WT) were established and tested for hypersensitivity against MMC. The increased sensitivity of *fan1-1* could be rescued in all four complementation lines. The fresh weight of the complementation lines was comparable to the fresh weight observed in WT plants (Figure 3B). Thus, the hypersensitivity of *fan1-1* against MMC is indeed due to mutation of the *FAN1* gene. The results sustain our conclusion that FAN1 plays a role in interstrand CL repair in *A. thaliana*.

### The VRR nuclease domain and the putative UBZ domain are essential for the function of AtFAN1 during interstrand CL repair

**Figure 4.**
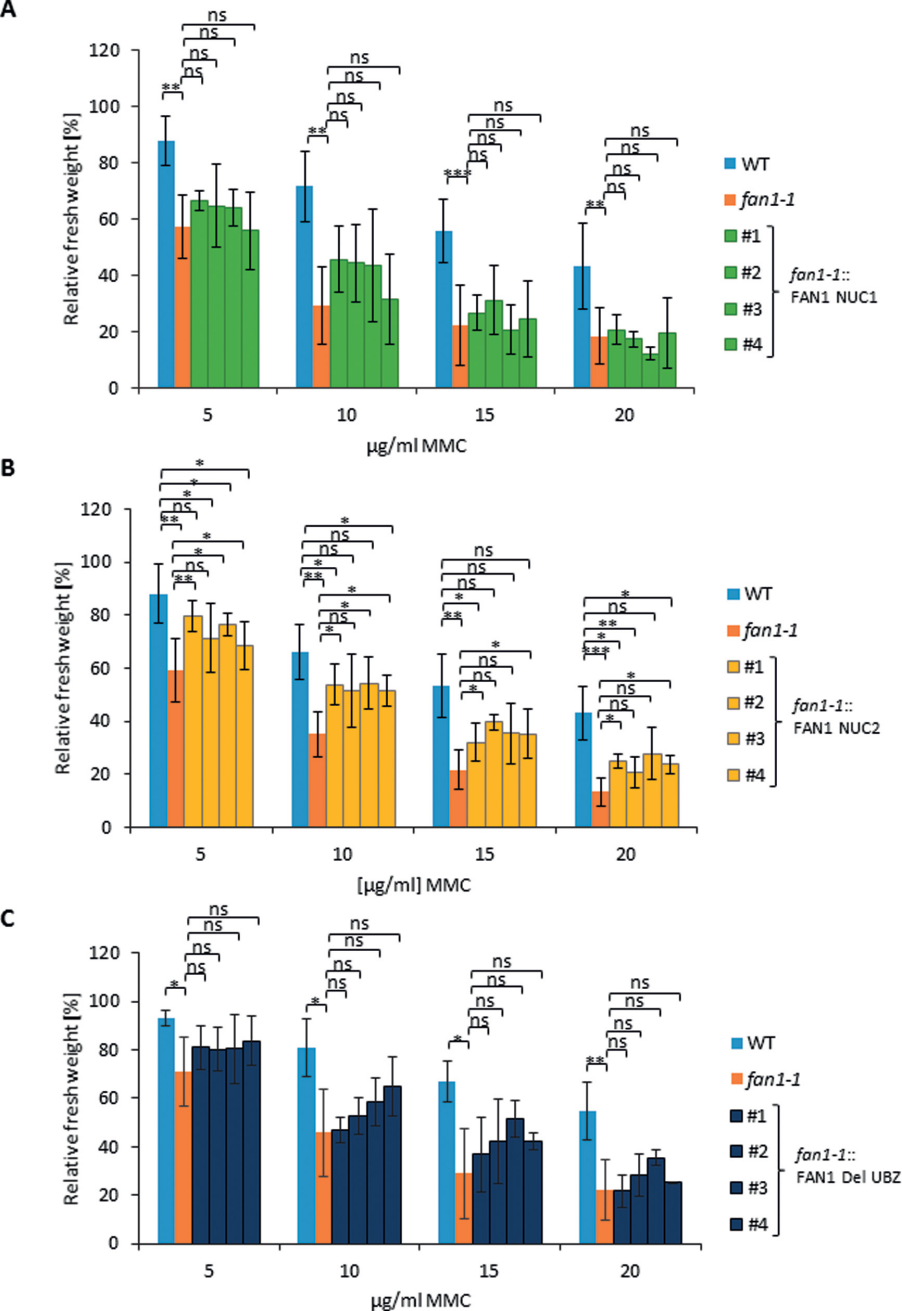
Contribution of FAN1 domains to the repair of MMC-induced DNA lesions. Sensitivity of the *fan1-1*::FAN1 NUC1, *fan1-1*::FAN1 NUC2 and the *fan1-1*::FAN1 Del UBZ complementation lines after MMC treatment. To calculate relative fresh weights of the tested lines, absolute fresh weights of MMC-treated plants were normalized with fresh weights of untreated control plants from identical lines. Each assay was performed at least three times to calculate mean values and standard deviations (error bars). (**A**) The complementation lines *fan1-1*::FAN1 NUC1 #1, #2, #3 and #4 showed a relative fresh weight comparable to that of the *fan1-1* mutant and were not able to complement the hypersensitivity of *fan1-1* against MMC. (**B**) The complementation lines *fan1-1*::FAN1 NUC2 #1, #2, #3 and #4 showed an intermediate relative fresh weight compared to WT plants and the *fan1-1* mutant. (**C**) The complementation lines *fan1-1*::FAN1 Del UBZ #1, #2, #3 and #4 showed a relative fresh weight comparable to the *fan1-1* mutant and were not able to complement the increased sensitivity of *fan1-1* against MMC. *P*-value ≤ 0.05 (*); *P*-value < 0.01 (**); *P*-value < 0.001 (***).

We were able to identify in Arabidopsis FAN1 a VRR nuclease domain and a putative UBZ domain. To analyze if these domains are essential for the CL repair function of FAN1 in Arabidopsis, we created different mutation or deletion constructs and analyzed whether they were able to complement the increased sensitivity against MMC observed in *fan1-1*. To analyze the VRR nuclease domain, we cloned two different constructs each carrying a point mutation to inhibit the nuclease activity of FAN1. These two point mutations have shown in humans to limit the endonuclease activity of FAN1 on branched DNA structures to different extents (5). To amplify these constructs, genomic DNA was used. Both constructs were under the control of the natural *FAN1* promoter and terminator. In the first construct, named FAN1 NUC1, the asparagine acid residue at position 833 was replaced by an alanine residue. In the second construct, named FAN1 NUC2, the lysine residue at position 854 was replaced by an alanine residue. In biochemical experiments with the human protein corresponding to the K854A mutant, some minor residual activity was detected. No activity was observed with a protein corresponding to a D833A mutant (see Supplementary Figure S3 in (5)). Both constructs were transformed into the *fan1-1* mutant line. Four independent *fan1-1*::FAN1 NUC1 and *fan1-1*::FAN1 NUC2 complementation lines were established before sensitivity against MMC was tested. The increased sensitivity of *fan1-1* could not be complemented by the FAN1 NUC1 construct; all tested complementation lines had the same fresh weight after MMC treatment as the *fan1-1* mutant (Figure 4A). Complementation lines carrying the FAN1 NUC2 construct exhibited an intermediate phenotype, as the hypersensitivity of *fan1-1* was complemented only partially (Figure 4B). These findings indicate that the K854A mutation retained some residual nuclease activity, as observed in mammals.

To test if the putative UBZ domain in AtFAN1 plays a role during FAN1 interstrand CL repair, we cloned a construct in which the entire potential UBZ domain from position 43 to 75 aa was deleted. This construct was named FAN1 Del UBZ. The construct was transformed into *fan1-1*. Following line establishment, four complementation lines were analyzed for sensitivity against MMC. The tested complementation lines could not complement the sensitivity phenotype of *fan1-1* after MMC treatment (Figure 4C). Therefore, the putative UBZ domain seems to be essential for the DNA repair function of FAN1 in plants.

We also tested the expression of *FAN1* in all complementation lines. The expression level of the gene was comparable or higher than the expression level of *FAN1* in WT plants (Supplementary Table S1).

### AtFAN1 is of special importance for the survival of meristematic cells in roots after the induction of CL DNA damage

We inferred that FAN1 is involved in the repair of interstrand CLs in *A. thaliana*. It is an interesting question whether such a protein is of similar importance to all types of cells in a multicellular organism. For this purpose, we analyzed root cells of the *fan1-1* mutant and WT plants stained with PI. Using PI, it is possible to label dead cells with a red fluorescent signal, since the labeling reagent is not able to pass through the membrane of living cells (46). The root meristem is a tissue of dividing cells. It is therefore suitable to analyze the effect of mutated genes on replication and dividing cells. It is composed of the stem cell niche and transiently amplifying (TA) cells. The stem cells (SCs) remain undifferentiated due to contact with the quiescent center (QC). SCs are located ∼5 to 6 cell layers above the root tip. The cells of the QC itself only rarely divide. SCs can be classified into the following four different types: epidermal SCs differentiate into cells of the epidermis or lateral root cap, endodermal SCs form the endodermis, columella SCs differentiate into central root cap cells and vascular SCs later form the leading tissue (47).

**Figure 5.**
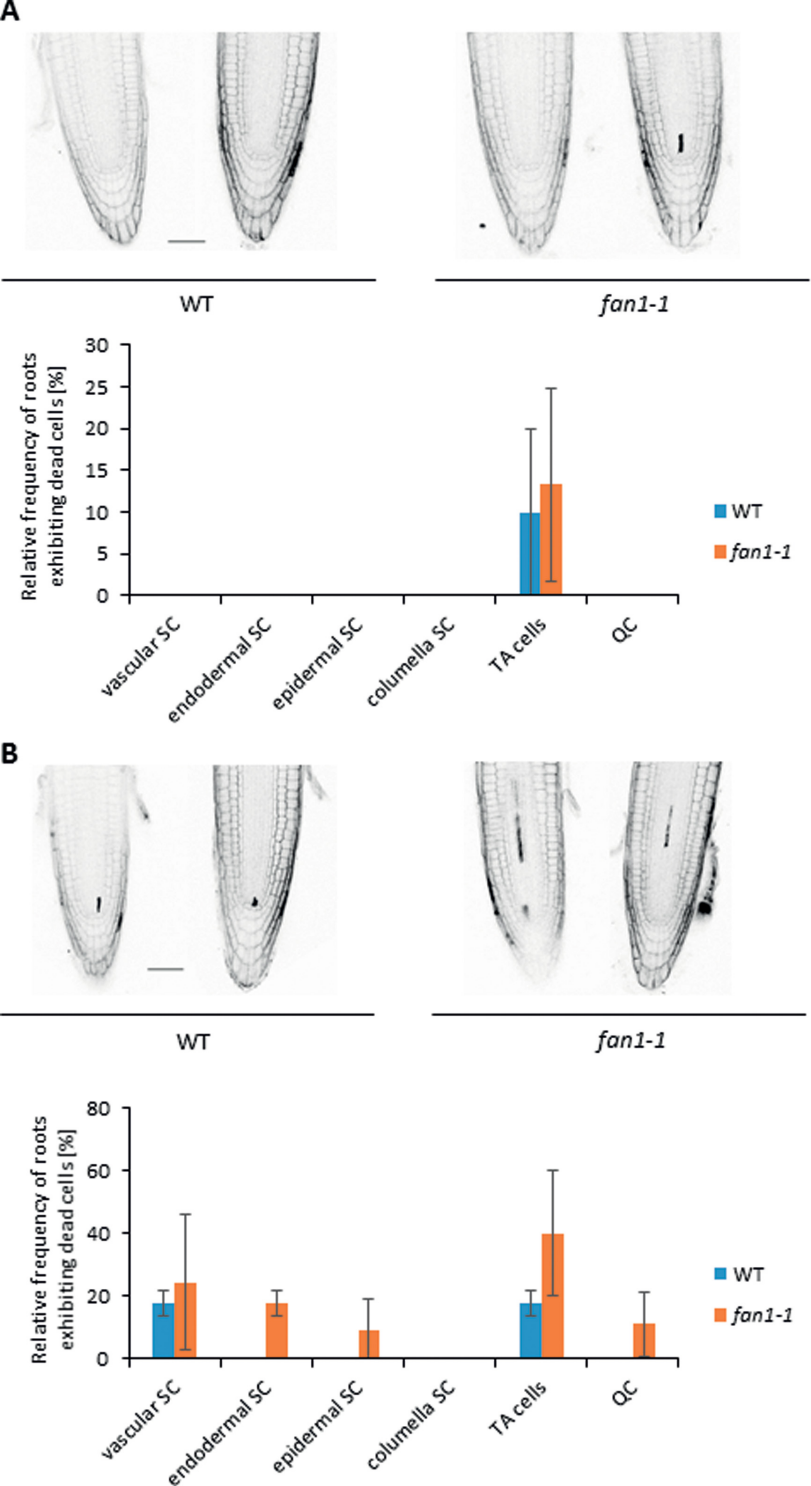
Analysis of dead cells in the root tips of *fan1-1* and WT plants. After staining with PI, the amount of whole root tips exhibiting at least one dead SC was determined. The SCs were differentiated into vascular SC, endodermal SC, epidermal SC, columella SC, TA cells and cells of the QC. (**A**) Representative images of confocal planes from PI-stained root tips of *fan1-1* and WT plants and the quantification of whole root tips with dead cells under normal conditions. (**B**) Representative images of confocal planes from PI-stained root tips of *fan1-1* and WT plants and the quantification of whole root tips with dead cells after treatment with 2.5 μg/ml MMC.

We determined the frequency of roots exhibiting at least one dead cell among the epidermal, endodermal, columella or vascular SCs, TA cells or QC. Roots from both *fan1-1* and WT plants showed ∼12% dead TA cells; dead cells were not found within the other cell types (Figure 5A). As we demonstrated that AtFAN1 plays a role in the repair of MMC-induced DNA damage, we wanted to test which types of root cells were harmed after MMC treatment. The roots of 5-day-old *fan1-1* and WT plants were incubated for 18 h in 2.5 μg/ml MMC. Plants were then stained with PI. As with the analysis for spontaneous replication damage, the frequency of roots with at least one dead SC was determined. The roots of *fan1-1* and WT plants exhibited a comparable amount of dead vascular SCs, ∼20% (Figure 5B). Twenty percent of WT roots showed at least one dead TA cell; the amount of dead TA cells observed in *fan1-1* was double that of the frequency in WT. In WT roots, dead cells were not observed in any other SC type. In *fan1-1*, however, we found roots with dead endodermal SCs (20%) and epidermal SCs (10%) and dead cells within the QC (10%). Thus, FAN1 is especially important for the survival of meristematic cells after CL induction.

### The role of AtFAN1 during somatic HR

In humans, FAN1 is involved in HR. It is speculated that this involvement is in the processing of 3′ ends of DNA breaks or in later steps of HR (5,7–8). No enhanced HR-mediated sister chromatid exchange was found in chicken (48). To test if AtFAN1 plays a role in somatic HR, we crossed *fan1-1* with the reporter line IC9 (43). This reporter line contains the *GUS* reporter gene separated into two non-functional parts. As both parts share a homologous segment, the *GUS* gene can be restored only after intermolecular recombination, most probably between sister chromatids. Recombination events can convert the substrate 5-bromo-4-chloro-3-indolyl glucuronide (X-GlucA) into a blue stain. Hence, every recombination event results in a quantifiable blue sector on the plant (49). As the recombination frequency of the mutant resembles the frequency of WT plants, FAN1 seems to have no direct function in somatic HR in plants. This is in contrast with mammals, but in line with results from chicken (Supplementary Figure S4).

### AtFAN1 acts in a pathway independent of the nuclease AtMUS81 in interstrand CL repair

After defining a function for FAN1 in CL repair in Arabidopsis, it was of special interest to define the specific pathways the protein is involved in. It has already been shown that the nuclease MUS81 and the ATPase RAD5A are required for interstrand CL repair in *A. thaliana*; however, these enzymes act in independent pathways (31,37,39). Furthermore, it was recently demonstrated that the Fanconi-associated histone-fold protein MHF1 and the RecQ helicase RECQ4A both play roles in the repair of interstrand CLs in Arabidopsis. These two proteins act in parallel pathways as well (24). To classify AtFAN1 within the known interstrand CL repair pathways, we created plants with mutations in *FAN1* and one of the following CL repair genes: *MUS81, RAD5A, RECQ4A* or *MHF1*.

**Figure 6.**
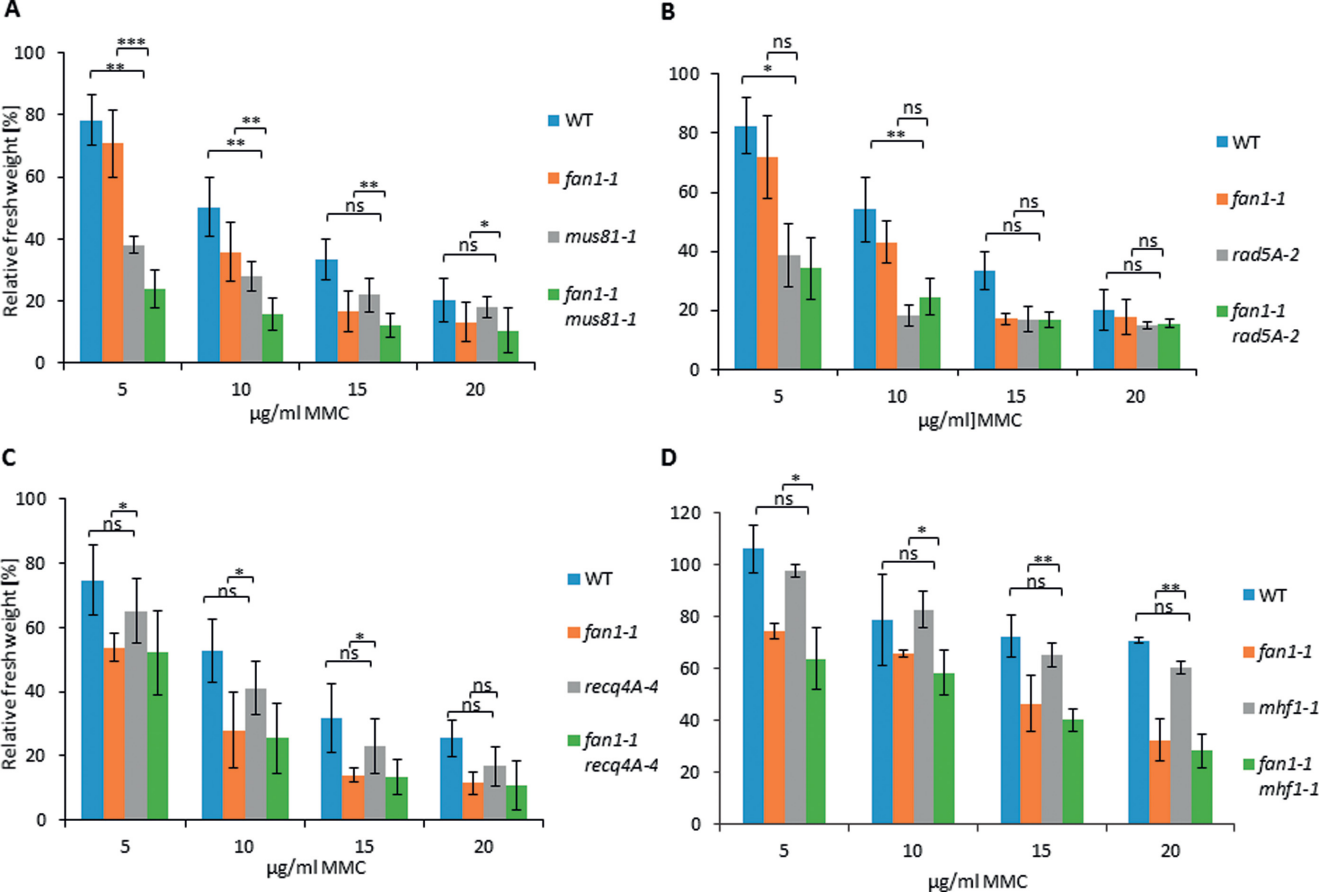
Sensitivity of different double mutants containing At*fan1-1* after MMC treatment. To calculate the relative fresh weights of the tested lines, the absolute fresh weights of MMC-treated plants were normalized with fresh weights of untreated control plants from identical lines. Each assay was performed at least three times to calculate the mean values and standard deviations (error bars). (**A**) The relative fresh weight of *fan1-1 mus81-1* was decreased compared to the relative fresh weigh of both single mutants. (**B**) The *fan1-1 rad5A-2* double mutant exhibited a relative fresh weight comparable to the fresh weight of the *rad5A-*2 single mutant. (**C**) The *fan1-1 recq4A-4* double mutant showed a relative fresh weight comparable to that of the *fan1-1* single mutant. (**D**) The relative fresh weight of the *fan1-1 mhf1-1* double mutant was similar to that of the *fan1-1* single mutant. *P*-value ≤ 0.05 (*); *P*-value < 0.01 (**); *P*-value < 0.001 (***). ns = not significant.

The *fan1-1* and *mus81-1* double mutant was created by crossing both single mutants. The homozygous double mutant was identified in the F2 generation through PCR-based genotyping. To characterize the sensitivity of *fan1–1 mus81-1* after MMC treatment, we performed sensitivity assays. *fan1-1 mus81-1* showed greater sensitivity than both single mutants after treatment with 5 and 10 μg/ml MMC; the relative fresh weight of the double mutant was significantly reduced compared to both single mutants (Figure 6A). Thus, the nucleases FAN1 and MUS81 seem to act in independent pathways during interstrand CL repair in *A. thaliana*.

### AtFAN1 shares common pathways with AtRECQ4A, AtRAD5A and AtMFH1 in interstrand CL repair

The *fan1-1 rad5A-2* double mutant was created by crossing the single mutants and identifying the homozygous double mutant through PCR-based genotyping. As with the analysis of *fan1-1 mus81-1*, we characterized the sensitivity of the *fan1-1 rad5A-2* double mutant by measuring the fresh weight after the treatment with different concentrations of MMC. The relative fresh weight of the double mutant *fan1-1 rad5A-2* was half of that of the *fan1-1* single mutant, but similar to the fresh weight of *rad5A-2* after treatment with 5 and 10 μg/ml MMC (Figure 6B). After treatment with 15 and 20 μg/ml MMC, both the single mutants and the double mutant had the same relative fresh weights compared to the untreated controls. As the double mutant resembled the increased sensitivity observed in the *rad5A-2* single mutant under treatment with lower concentrations of MMC, it is conceivable that FAN1 acts in the same pathway as the ATPase RAD5A in Arabidopsis.

It has been recently demonstrated that the RecQ helicase AtRECQ4A functions in the repair of interstrand CLs. This function is only detectable when both At*RECQ4A* and the Fanconi-associated gene At*MHF1* are mutated (24). To test if an additional mutation of the Fanconi-associated nuclease *FAN1* in the *recq4A* mutant line results in greater sensitivity compared to both single mutants as well, we created the *fan1-1 recq4A-4* double mutant line. The sensitivity of the double mutant was determined by measuring the fresh weight of *fan1-1 recq4A-4* after treatment with different concentrations of MMC. Under similar treatments, the fresh weights of the *fan1-1* and *recq4A-4* single mutants and the WT line were also determined. In contrast with the *mhf1 recq4A* double mutant, the *fan1-1 recq4A-4* double mutant showed no greater sensitivity than the *fan1-1* single mutant after MMC treatment (Figure 6C). As expected, the *recq4A-4* single mutant did not show an increased sensitivity against MMC compared to WT plants. These results indicate that AtFAN1 might play a role in the same pathway as AtRECQ4A during interstrand CL repair.

In humans, FAN1 and MHF1 are both associated with the FA pathway. In Arabidopsis, both of these proteins are also involved in the repair of interstrand CL repair. This finding prompted us to test an At*fan1-1* At*mhf1-1* double mutant. The double mutant was created by crossing both single mutants. PCR-based genotyping in the F2 generation revealed a double mutant homozygous for *fan1-1* and *mhf1-1*. The line was tested for sensitivity against interstrand CLs induced by MMC. As previously demonstrated (24), the *mhf1-1* single mutant did not exhibit hypersensitivity in comparison to WT plants (Figure 6D). However, the *fan1-1 mhf1-1* double mutant showed increased sensitivity against treatment with MMC similar to the *fan1-1* single mutant. As AtMHF1 must be involved in interstrand CL repair, the double mutant results suggest that AtFAN1 functions in a common pathway with AtMHF1 in the repair of interstrand CLs.

## DISCUSSION

The nuclease FAN1 is one of the repair factors that is activated and recruited by the FA pathway to repair interstrand CLs in humans. Recruitment to the DNA damage site is dependent on the interaction between monoubiquitinated FANCD2 and FAN1. In humans, FAN1 is involved in CL repair; however, its role cannot be generalized for all eukaryotes. Some organisms harbor no FAN1 homolog in their genomes. In others, FAN1 has no role in CL repair. Here, we demonstrated that the FAN1 homolog in the model plant *A. thaliana* is involved in interstrand CL repair. We also defined its role in relation to other DNA processing enzymes involved in DNA repair in plants.

### A single *FAN1* homolog is present in *A. thaliana*

Through BLAST analyses, a homolog of FAN1 could be detected in the Arabidopsis genome. FAN1 can also be found in other plant species, including monocotyledons and mosses. These findings indicate that there might be some selective advantage for plants to keep such a homolog. It is interesting that neither all fungi nor all animals harbor a FAN1 homolog in their genome. To gain further insight into the relationship of different FAN1 homologs in animals, plants and fungi, we calculated a phylogenetic tree. This tree showed that the relationship between FAN1 homologs in animals and plants is, unexpectedly, closer than that between animals and fungi. This is most likely because FAN1 homologs in animals and plants share a domain that is not conserved in fungi. In general, all FAN1 homologs are characterized by the presence of a VRR nuclease domain. We were able to locate a region within the open reading frame of plant homologs of FAN1 that might be able to represent a functional UBZ domain. This domain is present in mammalian homologs and absent in fungal homologs. This is likely because other factors in the FANC pathway are less conserved in fungi than in plants. For example, a FAN1 homolog is present in the genome of the fungus *S. pombe*. No obvious homologs of the FANCD2/I complex have been found in the *S. pombe* genome, though these proteins are conserved in Arabidopsis. In principle, the recruitment of FAN1 by a monoubiquitinated FANCD2/I complex might occur in plants using a mechanism similar to that in animals. As *S. pombe* lacks the respective factors involved in recruiting FAN1, the UBZ domain in FAN1 thought to be required for this recruiting step is obviously obsolete in such an organism.

### The *FAN1* homolog in *A. thaliana* is involved in interstrand CL repair

The analysis of the *FAN1* mutant *fan1-1* in *A. thaliana* revealed an increased sensitivity against the interstrand CL-inducing agent MMC; similar sensitivities have been observed in humans and in fission yeast. A detailed investigation of roots revealed that, in the absence of FAN1, treatment with CL-causing agents leads to replicative stress and finally cell death especially in the fast dividing SCs in the root meristem. Interestingly, FAN1 might not have a direct function in interstrand CL repair in all organisms. Depletion of *FAN1* in Xenopus egg extract does not seem to impair interstrand CL repair *in vitro* (23). Even though Xenopus FAN1 does not seem to be directly involved in interstrand CL repair, the possibility that it functions during interstrand CL repair in a redundant manner has not been excluded. FAN1 might have other functions, depending on the organism. Recently, mammalian FAN1 and the RecQ helicase BLM have been identified as factors involved in the recovery of stalled replication forks in a pathway independent of FANCD2 (50). We found no indication that FAN1 plays a general role in replication restart in root cells. In addition, we did not find any enhancement of cell death in meristematic root cells under standard growth conditions in the *fan1-1* mutant. The DNA helicase RTEL1 seems to play a major role in these types of processes in Arabidopsis. In the *rtel1* mutant background, as in the mus81 background, enhanced cell death in meristematic root cells was observed (51).

It was also reported that FAN1 plays a minor role in double-strand break repair by HR in human cells. This was postulated to be due to a role in end resection. We were not able to detect any defect in HR in Arabidopsis with the assay system applied in this study. In this assay, a functional marker gene is restored after interchromosomal HR that most likely occurs between sister chromatids. Similarly, no change in HR-mediated sister chromatid exchange was found in the *fan1* mutant in chicken (48). In different organisms, slight differences in the importance of different factors in DNA end resection during HR have been detected (52). Thus, FAN1 might play a more prominent role in resection in mammals than in other organisms.

### The VRR nuclease domain and the putative UBZ domain are essential for AtFAN1 function during interstrand CL repair

To test whether the VRR nuclease domain is essential for interstrand CL repair by FAN1 in Arabidopsis, we cloned two different constructs containing a point mutation in the nuclease domain. The constructs were then transformed into the *fan1-1* mutant line. Sensitivity against MMC was determined to see if the construct could complement hypersensitivity in *fan1-1*. The first construct, FAN1 NUC1, carries an alanine instead of an asparagine at position 833. This construct was not able to complement the increased sensitivity against MMC that occurs in *fan1-1*. A recently published crystal structure of the FAN1 homolog in *Pseudomonas aeruginosa* reveals that the corresponding asparagine is required for the coordination of both Mn^2+^ ions in the active center of the nuclease domain (11). The fact that this nuclease-defective construct was not able to complement the sensitivity of the mutant indicates that a functional nuclease is essential for the function of FAN1 during interstrand CL repair. Similar results were obtained in humans, where the corresponding point mutation was not able to rescue the sensitivity phenotype of *fan1* to interstrand CLs (5).

We also tested a second nuclease-deficient construct, FAN1 NUC2. This construct possessed an alanine instead of a lysine at position 854. The FAN1 NUC2 construct was also transformed into *fan1-1*. The crystal structure shows that this lysine is involved in the binding of a phosphate group of the DNA within the active center (11). The increased sensitivity of *fan1-1* against MMC was only complemented partially in all tested FAN1 NUC2 complementation lines. This is in line with an *in vitro* investigation of the biochemical properties of a correspondingly mutated human FAN1 protein, which revealed minor nuclease activity (5). We assume that the mutated protein still contains the two active metal ions in its active center and thus retains some minor activity. The mutant protein therefore can contribute to the repair of some, but not all, MMC-induced interstrand CLs.

In addition to the VRR nuclease domain, the putative UBZ domain seems to be essential for the interstrand CL repair function of FAN1. This domain is responsible for the recruitment of FAN1 to the site of DNA damage via monoubiquitinated FANCD2 in humans. If this domain is mutated in human FAN1, the localization of FAN1 to the damage site is prevented (5–8). Furthermore, the increased sensitivity of *fan1* against interstrand CLs cannot be complemented via a FAN1 protein without an intact UBZ domain (6). In AtFAN1, a UBZ domain was not found by domain search algorithms; however, we were able to identify several conserved amino acids in the potential region of the putative UBZ domain in AtFAN1 when compared to HsFAN1. We tested whether this region is involved in the interstrand CL repair function of FAN1 by cloning a deletion construct of FAN1named FAN1 Del UBZ. In this construct, the complete putative UBZ domain was removed. The sensitivity analysis of the tested FAN1 Del UBZ lines revealed that this construct was not able to complement the hypersensitivity of *fan1-1* against MMC. The expression of *FAN1* in those lines was comparable to that in WT plants. However, we cannot exclude that the deletion of the putative UBZ domain leads to a misfolded protein that is preferentially degraded. In such a case, no complementation could be achieved in any way. Even though such a scenario is possible, we favor the hypothesis that AtFAN1 possesses a UBZ domain that is indeed essential for the function of AtFAN1 during interstrand CL repair. Similar to the situation in humans, we speculate that AtFAN1 is localized to stalled replication forks via an interaction between the UBZ domain and monoubiquitinated FANCD2. This might be tested by further experiments, e.g. *in vitro* analysis of the FAN1 protein variants.

### Defining the role of AtFAN1 in different pathways of interstrand CL repair in Arabidopsis

The analysis of the *fan1-1* single mutant clearly demonstrated a function for FAN1 during interstrand CL repair in Arabidopsis. In a further step, we wanted to determine if FAN1 acts together with or independently of other known CL repair proteins in *A. thaliana*.

We combined the *fan1* mutant with mutants of four different genes that play roles in CL repair in Arabidopsis. These genes were AtRAD5A, AtRECQ4A, AtMHF1 and AtMUS81.

The ATPase AtRAD5A was recently shown to be involved in interstrand CL repair in a parallel pathway to the nuclease AtMUS81 (31,39). To investigate if FAN1 acts in the same pathway as RAD5A or independently of it, we analyzed the corresponding double mutant *fan1-1 rad5A-2* for increased sensitivity against MMC. The double mutant exhibited a greater sensitivity than the *fan1-1* single mutant. However, a hypersensitivity comparable to that of the *rad5A-2* single mutant was observed. Hence, FAN1 seems to be involved in the same pathway as RAD5A during interstrand CL repair in *A. thaliana*.

Even though the *recq4A-4* single mutant does not show an increased sensitivity against MMC, a function for the RecQ helicase RECQ4A during interstrand CL repair in Arabidopsis was recently demonstrated in an indirect way. The double mutant of *RECQ4A* and the FA-associated gene *MHF1* showed increased sensitivity against treatment with MMC, although neither of the single mutants were sensitive (24). This indicates the presence of several redundant pathways for the repair of MMC-induced DNA lesions. Only the loss of more than one pathway has a noticeable phenotype *in vivo*. Therefore, we tested both RECQ4A and MHF1 mutant lines in combination with the FAN1 mutant. The relative fresh weight of the double mutants was comparable to that of the *fan1-1* single mutant. It is tempting to speculate that the Fanconi-associated nuclease FAN1 acts before the RecQ helicase RECQ4A and MHF1, which define two putative sub-pathways for processing intermediates in interstrand CL repair. In contrast to the DNA repair phenotype of the previous double mutant lines, the double mutant *fan1-1 mus81-1* was more sensitive against MMC than both single mutants. We concluded that both nucleases act in independent interstrand CL repair pathways in Arabidopsis.

Our analysis revealed a complex picture concerning the role of FAN1. The most parsimonious assumption is that FAN1 acts at an early step in CL repair. It has been previously postulated that FAN1 can act as one of the nucleases that are involved in the initial incision step to unhook the CL from double-stranded DNA. The respective intermediates are further processed at later steps. To replicate across the excised CL site, sequence information is required. This information can be supplied by different mechanisms; one mechanism would be via HR. Factors involved in HR show strong sensitivities against interstrand crosslinking agents in plants (e.g. (53–55)). In contrast, such information can also be supplied by fork regression. The Arabidopsis protein RECQ4A has been shown to be able to regress replication forks *in vitro* (42). Finally, PRR can also be used. There are strong indications that AtRAD5A is involved in an error-free PRR pathway; this process is postulated to involve strand switching between homologous sequences (31). Alternatively, translesion polymerases might insert nucleotides at the lesion site that cannot be involved in base pairing due to the CL (56). It has been demonstrated for the yeast homologs of the FANC proteins FANCM and MHF1, ScMph1 and ScMhf1, that they function in a common pathway with the yeast homolog of AtRAD5A, ScRad5 (57). Furthermore, in fission yeast it has been shown that SpFml1 and SpFan1, the *S. pombe* homologs of FANCM and FAN1, both act epistatically in interstrand CL resolution and therefore might act in a common repair pathway as well (22). Thus, it is speculated that SpFml1, SpMhf1 and SpFan1 act in a common repair pathway with SpRad8. Our analysis of the Arabidopsis *fan1-1 mhf1-1* double mutant suggests that both proteins are involved in the same pathway during interstrand CL repair. This phenomenon is similar to the situation in *S. pombe*: AtFAN1, AtMHF1 and AtRAD5A might also function in a common canonical interstrand CL repair pathway. It will be important to perform further experiments to support this hypothesis.

A number of different structure-specific nucleases might be involved in the initial unhooking step of the repair reaction, where the incision occurs at both sides of the CL. Several different candidates in humans have been discussed in the literature including FAN1, ERCC1/XPF, MUS81/EME1, SLX4/SLX1, SNM1A and SNM1B (58). The situation is even more complex, as these nucleases might complement for each other to a certain extent. These proteins might also be involved in later steps of the process, e.g. the final elimination of the CL from the non-replicated strand. Moreover, no clear homologs of the SLX4/SLX1 complex and of SNM1A and SNM1B have been have been identified in the Arabidopsis genome so far. Thus, we cannot draw a complete picture of nuclease-mediated CL repair in plants. We demonstrated here that FAN1 has an important function in CL repair; however, some of its functions may overlap with a second nuclease. The stronger sensitivity observed in the *RAD5A* mutant compared to the *FAN1* mutant indicates such a situation.

**Figure 7.**
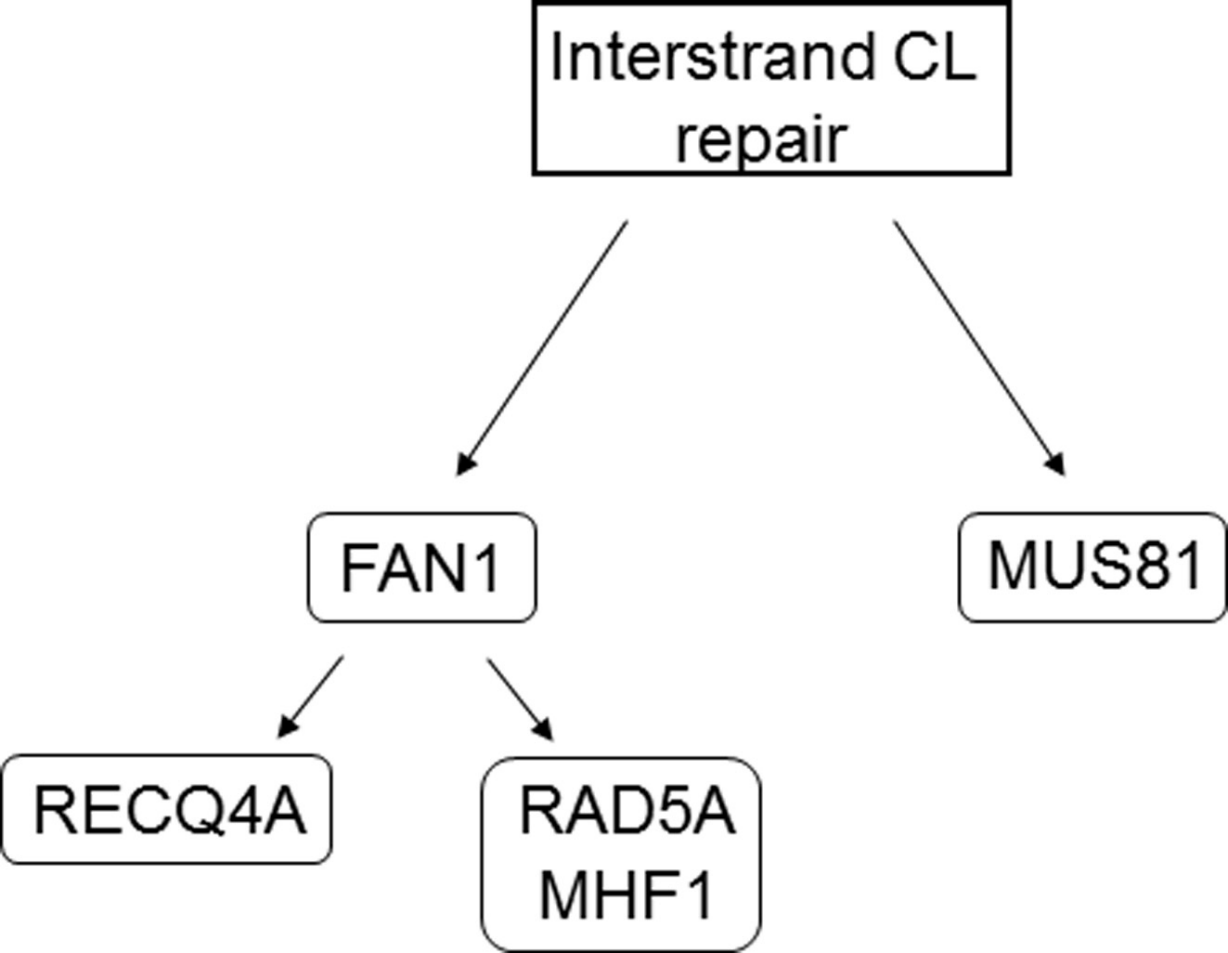
Model of different interstrand CL repair pathways with *fan1-1* in *A. thaliana*. During interstrand CL repair, FAN1 acts above the two sub-pathways defined by RECQ4A and RAD5A. We assume that MHF1 and RAD5A act in the same pathway. Furthermore, MUS81 defines a FAN1-independent ‘backup’ pathway of CL repair in plants.

We show that, in Arabidopsis, at least two of the nucleases tested in this study are not epistatic. These nucleases are involved in different CL repair pathways. MUS81 and FAN1 differ in their incision specificities; however, these two enzymes are able to process similar DNA structures, such as nicked Holliday junctions, although to different products. It has been speculated that one nuclease could be responsible for an incision on the 5′ DNA strand of the interstrand CL lesion and a second nuclease incises the 3′ strand of the CL. Therefore, FAN1 and MUS81 could work in a common pathway. In Arabidopsis, this seems not to be the case. In contrast, FAN1 might act as one of the incision nucleases in the first step of the canonical CL pathway. In this scenario, MUS81 is responsible for the processing of more complex DNA structures or intermediates that cannot be processed by other nucleases involved in the CL pathways. Our previous studies of MUS81 indicate that this nuclease is involved in the removal of replicative DNA intermediates that would otherwise block replication. This is also documented by the fact that the double mutants of *MUS81* and either *RECQ4A* or *FANCM* result in synthetic lethality phenotypes (24,32,59). If certain types of DNA damage cannot be removed by the action of specific sophisticated DNA helicases, the MUS81 nuclease might act as a safeguard and a universal tool to remove all intermediates that would otherwise result in dead ends. A model of how Arabidopsis FAN1 might work in interstrand CL repair in relation to all other factors tested in this study is shown in Figure 7.

The elucidation of DNA CL repair mechanisms is especially interesting from a plant perspective. Plants have, in contrast to mammals, beside the primary metabolism, a wealth of pathways of the secondary metabolism that are required for their survival in an adverse and changing environment and to combat pathogens. Many diverse aldehydes that have the capacity to form adducts or CLs with nuclear DNA are produced within these pathways. Therefore, it is tempting to speculate that plants require several CL repair pathways so that efficient repair is guaranteed, also in case of a high CL burden.

## SUPPLEMENTARY DATA

Supplementary Data are available at NAR Online.

SUPPLEMENTARY DATA
