# Supplementary material for: The nuclease FAN1 is involved in DNA crosslink repair in Arabidopsis thaliana independently of the nuclease MUS81
Source: Nucleic Acids Res. 2015 Mar 16;43(7):3653–66. doi: 10.1093/nar/gkv208 (PMC4402529; doi:10.1093/nar/gkv208)
Supplement: SUPPLEMENTARY DATA [file supp_43_7_3653__index.html]

The nuclease FAN1 is involved in DNA crosslink repair in Arabidopsis thaliana independently of the nuclease MUS81 — The nuclease FAN1 is involved in DNA crosslink repair in Arabidopsis thaliana independently of the nuclease MUS81 — SUPPLEMENTARY DATA 

# The nuclease FAN1 is involved in DNA crosslink repair in *Arabidopsis thaliana* independently of the nuclease MUS81

## SUPPLEMENTARY DATA

**Files in this Data Supplement:**

- SUPPLEMENTARY DATA
