# Supplementary material for: The nuclease FAN1 is involved in DNA crosslink repair in Arabidopsis thaliana independently of the nuclease MUS81
Source: Nucleic Acids Res. 2015 Mar 16;43(7):3653–66. doi: 10.1093/nar/gkv208 (PMC4402529; doi:10.1093/nar/gkv208)
Supplement: SUPPLEMENTARY DATA [file supp_gkv208_nar-00316-d-2015-File009.pdf]

Figure S1

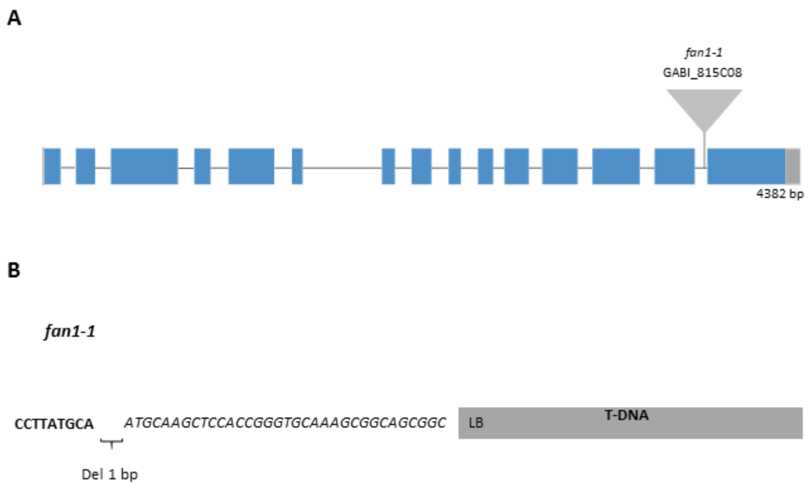

Supplementary Figure S1. Characterization of the At*FAN1* mutant line *fan1-1*. (A) The *fan1-1* (GABI_815C08) insertion site is located in intron 14. (B) Detailed analysis of the *fan1-1* insertion site. Genomic sequences are shown in bold; insertions are shown in italic. Deletions are abbreviated as Del, and their specific length is given.

Figure S2

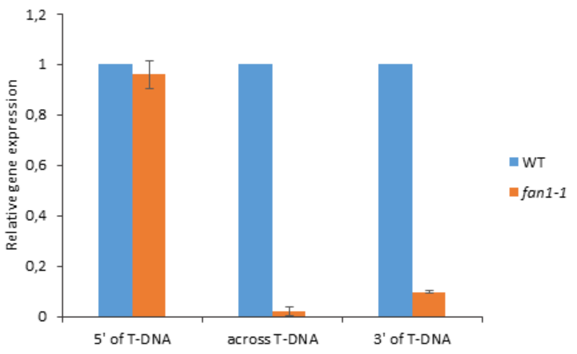

Supplementary Figure S2. Expression level of At*FAN1* in the *fan1-1* mutant line. In *fan1-1*, the relative gene expression in the 5’ region relative to the T-DNA insertion site is comparable to the expression in WT plants. Across the insertion site and in the 3‘ region, expression of *FAN1* significantly reduced or eliminated.

Figure S3

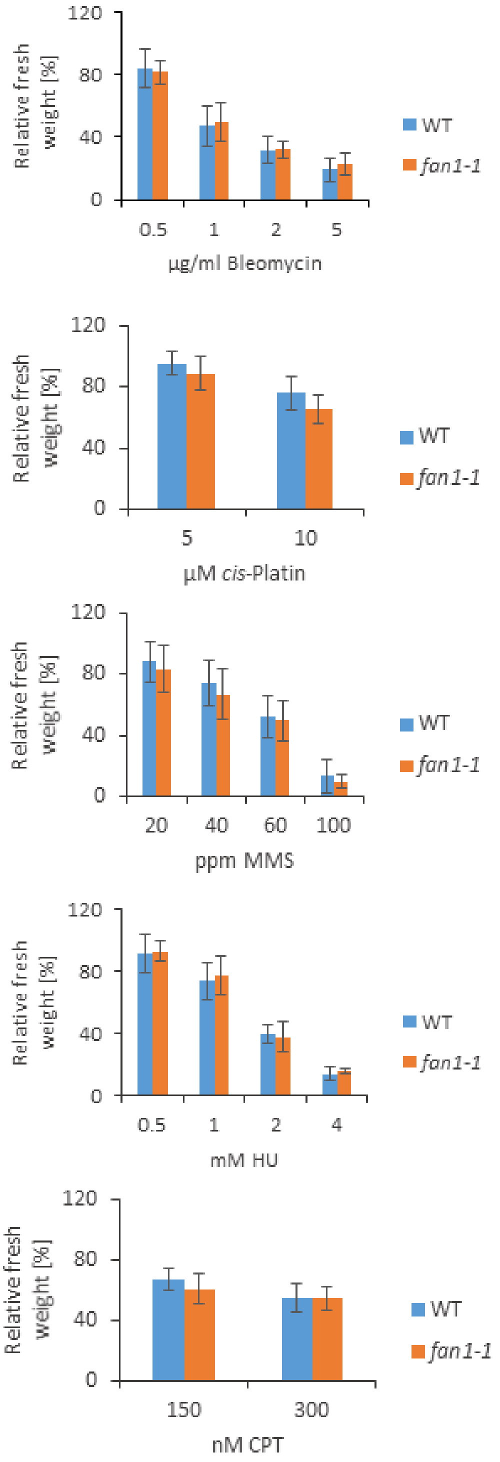

Supplementary Figure S3. Relative fresh weight of At*fan1-1* after treatment with bleomycin, *cis*-Platin, methylmethane sulfonate (MMS), hydroxyurea (HU) and camptothecin (CPT). To calculate relative fresh weights of the tested lines, absolute fresh weights of MMC-treated plants were normalized with fresh weights of untreated control plants from identical lines. Each assay was performed at least three times

to calculate the mean values and standard deviations (error bars). None of the genotoxin treatments resulted in an increased sensitivity of *fan1-1* compared to WT plants.

Figure S4

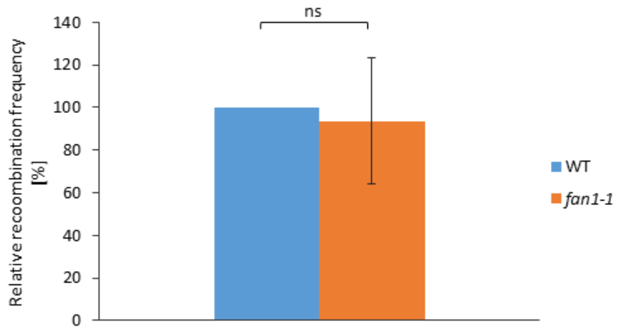

Supplementary Figure S4. Spontaneous somatic homologous recombination (HR) frequency of At*fan1-1*. The spontaneous HR frequency of *fan1-1* was calculated as the HR frequency normalized to the HR frequency of WT plants. The assay was performed at least three times to calculate mean values and standard deviations (error bars). At*fan1-1* showed no difference in spontaneous somatic HR frequency compared to WT plants. ns = not significant.

Supplementary Table S1. Expression levels of At*FAN1* in *fan1-1*::FAN1 WT (A), *fan1-1*::FAN1 NUC1 (B), *fan1-1*::FAN1 NUC2 (C) and *fan1-1*::FAN1 Del UBZ (D) complementation lines. *FAN1* expression in the lines containing the construct FAN1 NUC1, FAN1 NUC2 or FAN1 Del UBZ was similar or greater compared to the expression in WT plants.

Table S1

A

	**WT**	***fan1-1***	***fan1-1*::FAN1 WT**			
			**#1**	**#2**	**#3**	**#4**
**Replicate 1**	1	0,09	1,11	1,61	2,94	3,05
**Replicate 2**	1	0,08	4,11	4,92	3,23	3,62
**Replicate 3**	1	0,20	3,93	5,31	5,19	3,39
**Replicate 4**	1	0,04	0,95	1,90	1,55	2,73

B

	**WT**	***fan1-1***	***fan1-1*::FAN1 NUC1**			
			**#1**	**#2**	**#3**	**#4**
**Replicate 1**	1	0,04	19,42	70,17	81,78	69,17
**Replicate 2**	1	0,03	22,79	79,14	63,11	69,08
**Replicate 3**	1	0,13	24,15	61,84	45,07	43,28

C

	**WT**	***fan1-1***	***fan1-1*::FAN1 NUC2**			
			**#1**	**#2**	**#3**	**#4**
**Replicate 1**	1	0,04	6,00	9,21	36,15	73,17
**Replicate 2**	1	0,03	5,48	5,25	29,59	153,41
**Replicate 3**	1	0,13	4,14	4,08	27,71	173,82

D

	**WT**	***fan1-1***	***fan1-1*::FAN1 Del UBZ**			
			**#1**	**#2**	**#3**	**#4**
**Replicate 1**	1	0,23	2,05	0,42	3,66	1,47
**Replicate 2**	1	0,22	1,73	1,96	2,92	1,22
**Replicate 3**	1	0,22	2,19	2,06	4,19	1,47
**Replicate 4**	1	0,15	1,57	1,71	2,81	1,10
